# The potential effects and mechanisms of hispidulin in the treatment of diabetic retinopathy based on network pharmacology

**DOI:** 10.1186/s12906-022-03593-2

**Published:** 2022-05-19

**Authors:** Yao Chen, Jiaojiao Sun, Zhiyun Zhang, Xiaotong Liu, Qiaozhi Wang, Yang Yu

**Affiliations:** 1 Department of Histology Anatomy and HistoEmbryology, School of Basic Medical Sciences, Southwest Medical University, Luzhou, Sichuan 646000 People’s Republic of China; 2 Key Laboratory of Medical Electrophysiology of Ministry of Education and Medical China, Luzhou, Sichuan 646000 People’s Republic of China; 3 Electrophysiological Key Laboratory of Sichuan Province, Institute of Cardiovascular Research, Southwest Medical University, Luzhou, Sichuan 646000 People’s Republic of China; 4 Department of Clinical Medicine, School of Clinical Medicine, Southwest Medical University, Luzhou, Sichuan 646000 People’s Republic of China; 5 Jiangyang City Construction College, Luzhou, Sichuan 646000 People’s Republic of China

**Keywords:** Diabetic retinopathy, *Eriocauli Flos*, Hispidulin, Network pharmacology, Molecular docking

## Abstract

**Background:**

Diabetic retinopathy (DR), one of the most common and severe microvascular complication of diabetes mellitus (DM), is mainly caused by diabetic metabolic disorder. So far, there is no effective treatment for DR. Eriocauli Flos, a traditional Chinese herb, has been used in treating the ophthalmic diseases including DR. However, the active ingredients and molecular mechanisms of Eriocauli Flos to treat diabetic retinopathy remain elusive.

**Methods:**

Here, the systems pharmacology model was developed via constructing network approach. 8 active components which were screened by oral bioavailability (OB ≥ 30%) and drug-likeness (DL ≥ 0.18) and 154 targets were selected from Eriocauli Flos through TCMSP database. Another 3593 targets related to DR were obtained from Genecards, OMIM, TTD, and Drugbank databases. The 103 intersecting targets of DR and Eriocauli Flos were obtained by Draw Venn Diagram. In addition, protein-protein interaction network was established from STRING database and the compound-target network was constructed by Cytoscape which screened top 12 core targets with cytoNCA module. Then the overlapping targets were analyzed by GO and KEGG enrichment. Moreover, two core targets were selected to perform molecular docking simulation. Subsequently, CCK8 assay, RT-PCR and Western blotting were applied to further reveal the mechanism of new candidate active component from Eriocauli Flos in high glucose-induced HRECs.

**Results:**

The results showed that the overlapping targets by GO analysis were enriched in cellular response to chemical stress, response to oxidative stress, response to reactive oxygen species, reactive oxygen species metabolic process and so on. Besides, the overlapping targets principally regulated pathways such as AGE-RAGE signaling pathway in diabetic complications, lipid atherosclerosis, fluid shear stress and atherosclerosis, and PI3K-Akt signaling pathway. Molecular docking exhibited that VEGFA and TNF-α, had good bindings to the great majority of compounds, especially the compound hispidulin. In vitro, hispidulin ameliorated high-glucose induced proliferation by down-regulating the expression of p-ERK, p-Akt, and VEGFA; meanwhile inhibited the mRNA levels of TNF-α.

**Conclusions:**

In this study, through network pharmacology analysis and experimental validation, we found that hispidulin maybe has a potential targeted therapy effect for DR by decreasing the expression of p-Akt, p-ERK, and VEGFA, which resulted in ameliorating the proliferation in HRECs.

**Supplementary Information:**

The online version contains supplementary material available at 10.1186/s12906-022-03593-2.

## Introduction

Diabetic retinopathy (DR) is a common complication of diabetes with microvascular lesions that is featured by vascular endothelial injury including pericyte loss, endothelial cell proliferation, basement membrane thickening, retinal ischemia and hypoxia, and angiogenesis [1, 2]. The major clinical pathogenesis now is mainly related to the activation of protein kinase C, the accumulation of terminal glycosylation products, and the hyperactivity of polyol pathway which may ultimately induce inflammatory reactions with upregulation of vascular endothelial growth factor (VEGF), tumor necrosis factor α (TNF-α), angiopoietin − 2 (Ang-2) and intercellular adhesion molecule-1 (ICAM-1), as well as result in the destruction of the blood-retinal barrier (BRB) and impairment of retinal vascular function [3, 4]. VEGFA is one of the potent pro-angiogenic factor that links angiogenesis and inflammation, which induces the proliferative changes associated with neovascularization in the progression of DR [5, 6]. More importantly, high glucose significantly increases the protein expression levels of VEGFA [7], and thereby, anti-VEGF therapy currently still serves as a prominent role in the management of DR  [8]. *Eriocauli Flos*, a Chinese herbal medicine, is traditionally used in ophthalmic diseases with the function of coursing wind, dissipating heat and improving eyesight in the theory of traditional Chinese medicine [9]. The ingredients of *Eriocauli Flos* mainly belong to flavono, lavones, isoflavones, xanthones, naphthopyranones and phenolic acids, with pharmacology effects including anti-inflammatory and anti-bacterial [10, 11]. Hispidulin (Dinatin), one of the flavonoid compounds from *Eriocauli Flos,* has been reported had the anti-inflammatory, anti-cancer, anti-oxidative stress effects [12]. For example, hispidulin inhibited HCC cell proliferation and metastasis by activating of peroxisome proliferator-activated receptor γ (PPARγ ) [13] and another study demonstrated that hispidulin suppressed allergic inflammatory reaction by reducing histamine release and inflammatory cytokines such as tumor necrosis factor-α (TNF-α) and interleukin-4 (IL-4 ) [14]. However, whether hispidulin has effects on high glucose-induced in vitro diabetic retina model by using HRECs cells is unclear.

Network pharmacology, combining system biology with computer technology, systematically observes the influence of drugs on diseases through network data analysis on the basis of “drug target of disease gene” interaction, reveals the molecular mechanism of drug action on diseases, as well as provides new ideas and directions for new drug research and development [15, 16]. Molecular docking, a method of simulating the interactions between proteins and small molecules, has become an important technology in the field of computer aided drug research [17, 18]. Therefore, the network pharmacology method was used to explore the targets, biological function and pathways of *Eriocauli Flos* against DR. Then, hispidulin was screened as the most effective compound from *Eriocauli Flos* by molecular docking. Furthermore, the effects of hispidulin against DR was further investigated by quantitative RT-PCR and Western blotting to test the mRNA and protein expression levels of p-Akt, p-ERK, and VEGFA. This study provided the theoretical foundation and laid the theoretical foundation for the subsequent experiment validation.

## Material and methods

### Material

Hispidulin (1447-88-7) was purchased from Victory Biological Technology, China and D-glucose (G8270) was from Sigma, USA.

### Screening active ingredients of *Eriocauli Flos*

All the compounds of *Eriocauli Flos* were gathered by using Traditional Chinese Medicine Systems Pharmacology Database (TCMSP) (http://sm.nwsuaf.edu.cn/lsp/tcmsp.phpphp), [19] and the active ingredients were filtered by oral bioavailability (OB ≥ 30%) and drug-likeness (DL ≥ 0.18) [20]. The targets ‘s information of the active ingredients were also obtained from TCMSP and then affirmed through UniProt database (https://www.uniprot.org/ ) [21] to be further analyzed.

### Obtaining targets related to DR and intersection targets of *Eriocauli Flos* and DR

The targets associated with DR were predicted via GeneCards (https://www.genecards.org/), [22] Online Mendelian Inheritance in Man (OMIM) database (https://omim.org/), [23] Therapeutic Targets Database (TTD) (http://bidd.nus.edu.sg/group/ttd/ttd.asp) [24] and DrugBank database (https: www.drugbank.ca) [25]. All targets were searched by key words of “diabetic retinopathy” and set specie as “homo sapiens”. At last, the total targets of DR and Intersection targets of *Eriocauli Flos* and DR were displayed by Draw Venn Diagram (http://bioinformatics.psb.ugent.be/webtools/Venn/) [26].

### +Constructing “compound-target” network

To better acknowledge the interaction relationship between compounds and targets, the 8 active compounds and 103 intersection targets of *Eriocauli Flos* and DR were constructed into Cytoscape 3.8.0 to construct and analyze the network [27]. Each node represents a compound or a target, and each line represents relationship between the active ingredients and targets.

### Constructing protein-protein interaction (PPI) network of intersection targets of *Eriocauli Flos* and DR and screening key targets

Using STRING11.0 database (https://string-db.org/) [28] to predict the protein-protein interaction of 103 intersection targets of *Eriocauli Flos* and DR. To build the interaction network, minimum required interaction score > 0.9 was set and disconnected nodes in the network were hidden and 89 targets were obtained. Each node represents a protein and its structure, and each edge represents the associations between the different protein. And the PPI results were output as a TSV file which was then imported to Cytoscape 3.8.0 for further network analysis by using the cytoNCA plug-in, [29] and each filer got one score according to the degree, betweenness and closeness centrality. Degree is the most direct indicator to measure node centrality in network analysis and the higher the degree of a node reflects the more important it is in the network. Betweenness refers to the number of times that one node acts as the shortest bridge with two other nodes and the higher the number of times a node serves as an intermediary indicates the higher score of betweenness is. Closeness describes the average length of the shortest circuit of each node to other nodes and the closer a node is to other nodes means the higher score of closeness it is [30]. Hence, 12 key targets were screened by the condition of the median value greater than that of three parameters in the score.

### Gene ontology (GO) and the Kyoto encyclopedia of genes and gnomes (KEGG) enrichment analysis of the intersection targets

GO database (http://geneontology.org/), [31] including biological process, cell component, and molecular function terms, was used to describe gene functions and the results finally were input to excel for analysis. KEGG (www.kegg.jp/kegg/kegg1.html), [32] a knowledge database, was used to identify biological information and systematic function of target genes through obtaining significantly enriched biological pathways. Bioconductor clusterProfiler, an R package, was used to perform both enrichment analysis above by gene cluster [33].

### Molecular docking

The 2D structures of ligands (compounds) were downloaded from Pubchem database (https://pubchem.ncbi.nlm.nih.gov/) [34] and TCMSP database which then were imported to Chemoffice 2014 software for the sdf format switching to mol2 format (3D structure)**.** The 3D structures of proteins (targets) were obtained from RCSB PDB database (http://www.rcsb.org/pdb/) [35] and were input to Pymol software to separate the original ligand out and remove the hydrone, phosphate radical and other inactive ligands from the proteins [36]. The 3D structures of small molecules (ligands) and target proteins were imported into Auto Dock Vina software to acquire PDBQT format, and finally docking the molecules with the target proteins to observe the affinity and the hydrogen bond interaction was shown by using pymol [37].

### Cell culture

Human retinal endothelial cells (HRECs) were obtained from BNCC and the cells previously were grown in Dulbecco’ Modified Eagle’s Medium (C11995500BT, Gibco, USA) supplemented with 10% fetal calf serum (10,270,106, Gibco, USA) and 1% (v/v) penicillin–streptomycin (SV30010, HyClone, China) at 37 °C in 5% CO2 incubator. For the hispidulin treatment study, HRECs were exposed to normal glucose (NG, 5 mmol/L) and high glucose (HG,30 mmol/L) with or without hispidulin for 48 h according to previous research about high glucose model establishment in HRECs. All the cells in the experiment were used from 3 to 6 passages.

### HRECs viability assay

The viability of HRECs was detected by CCK-8 kit (C0038, Beyotime, China). Briefly, 8 × 10^3^ cells per well were seeded into 96- well plate and cells were exposed to normal glucose (5 mM) with or without hispidulin (5-80 μM) for 24 h to assess cytotoxicity. Then 5 × 10^3^ cells per well were seeded into 96- well plate and cells were treated with glucose (5 and 30 mM) with or without different concentrations of hispidulin (5-40 μM) for 48 h after the cells adhered for 24 h, Cell viability was measured following the manufacturer’s instructions.

### RNA extraction and quantitative real-time polymerase chain reaction (RT-qPCR)

For the quantitative real-time PCR of VEGFA and TNF-a, 2.0× 10^5^ HRECs were seeded into six-well plates. After treatment with Hispidulin for 48 h, total RNA was extracted using Trizol reagent (15596–026, Invitrogen, USA) and was reverse-transcribed into cDNA with ReverTra Ace qPCR RT Master Mix (FSQ-201, TOYOBO, Japan). Finally, 2 μl cDNA was used for fluorescence quantitative PCR analysis by using SYBR Green Realtime PCR Master Mix (QPK-201, TOYOBO, Japan). The relative CT method (△△CT) was used to calculate the relative transcriptome level, and the normalized target protein number was compared with the endogenous reference gene GAPDH. The specific primers used were as follows: human TNF-α (forward,5′-AGGCGCTCCCCAAGAAGACAG-3′; reverse,5′-AGCAGGCAGAAGAGAGCGTGGTG-3′), human VEGFA (forward,5′-GAACTTTCTGCTGTCTTGGG-3′;reverse,5′-CTTCGTGATGATTCTGCCC-3′), humanGAPDH (forward,5′-CAATGACCCCTTCATTGACC-3′;reverse,5′-GACAAGCTTCCCGTTCTCAG − 3′).

### Western blotting

The cells were lysed with RIPA lysate (P0013C, Beyotime, China) containing a protease inhibitor, phosphatase inhibitor and PMSF. Then, lysates were centrifuged at 14,000 rpm, for 10 min at 4 C, and the supernatants were extracted, and resuspended in the 5× loading buffer (P0015, Beyotime, China) to prepare the protein samples. The electrophoresis was performed on SDS-PAGE and then the proteins were transferred to PDVF membranes (ISEQ00010, Millipore, USA). Following blocked of the membranes with 5% skim milk for 1 h and the membranes were then incubated overnight at 4 °C with primary antibodies as follows: VEGFA (ab214424, abcam, China), ERK (4695, Cell Signaling Technology, USA), p-ERK (4370, Cell Signaling Technology, USA), Akt (bsm-33,278 M, Bioss, China) and p-Akt (bsm-33,281 M, Bioss, China). Then membranes were incubated by the addition of a secondary antibody for 1 h at room temperature after another three times wash with TBST. Finally, the protein expression was detected by using ECL chemiluminescence substrate (P0018S, Beyotime, China).

### Statistical analysis

All data were shown as mean ± SD and analyzed using one-way ANOVA and followed by Dunnett’ s multiple comparison test. *P* < 0.05 was considered as statistically significant.

## Results

### Collection of targets of *Eriocauli Flos* and DR

**Fig. 1:**
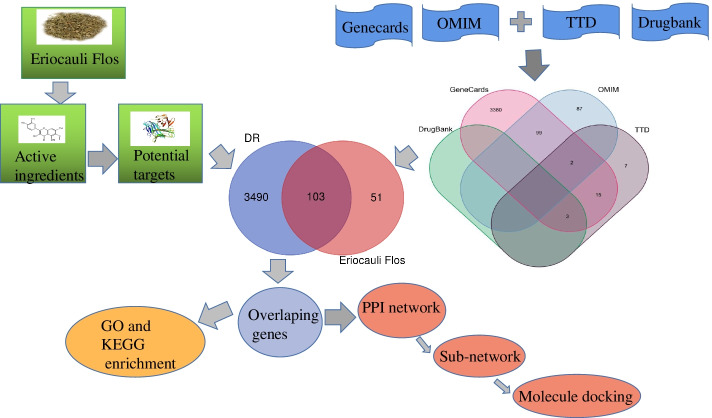
Scheme design of network pharmacological data analysis

A total of 8 compounds of *Eriocauli Flos* from TCMSP database, containing 154 targets, reached the criteria of OB ≥ 30% and DL ≥ 0.18. The goals (Table 1) including MOL001735 (Dinatin), MOL002281 (Toralactone), MOL002721 (Quercetagetin), MOL004112 (Patuletin), MOL006425 (2-benzo [1, 3] dioxol-5-yl-5,7-dimethoxy-chroman), MOL006426 (1,3,6-trihydroxy-2,5,7-trimethoxyxanthone), MOL006428 (7,30-dihydroxy-5,40,50-trimethoxyisoflavone) and MOL000098 (Quercetin). 3593 targets of diabetic retinopathy were obtained from Genecards, OMIM, TTD and Drugbank database, and 103 intersection targets were ultimately filtered from the targets of *Eriocauli Flos* and DR that the specific information were displayed by venn diagram in the Fig. 1**.**

**Table 1:** Detailed information of effective compounds which were screened by oral bioavailability (OB ≥ 30%) and drug-likeness (DL ≥ 0.18) from *Eriocauli Flos*

Number	Mol ld	Molecule name	OB %	DL
1	MOL001735	Dinatin (Hispidulin)	30.97	0.27
2	MOL002281	Toralactone	46.46	0.24
3	MOL002721	Quercetagetin	45.01	0.31
4	MOL004112	Patuletin	53.11	0.34
5	MOL006425	2-benzo [1, 3] dioxol-5-yl-5,7-dimethoxy-chroman	57.68	0.38
6	MOL006426	1,3,6-trihydroxy-2,5,7-trimethoxyxanthone	42.22	0.38
7	MOL006428	7,30-dihydroxy-5,40,50-trimethoxyisoflavone	47.33	0.37
8	MOL000098	Quercetin	46.43	0.2

### Network between compounds and intersection targets of Eriocauli Flos and DR

**Fig. 2:**
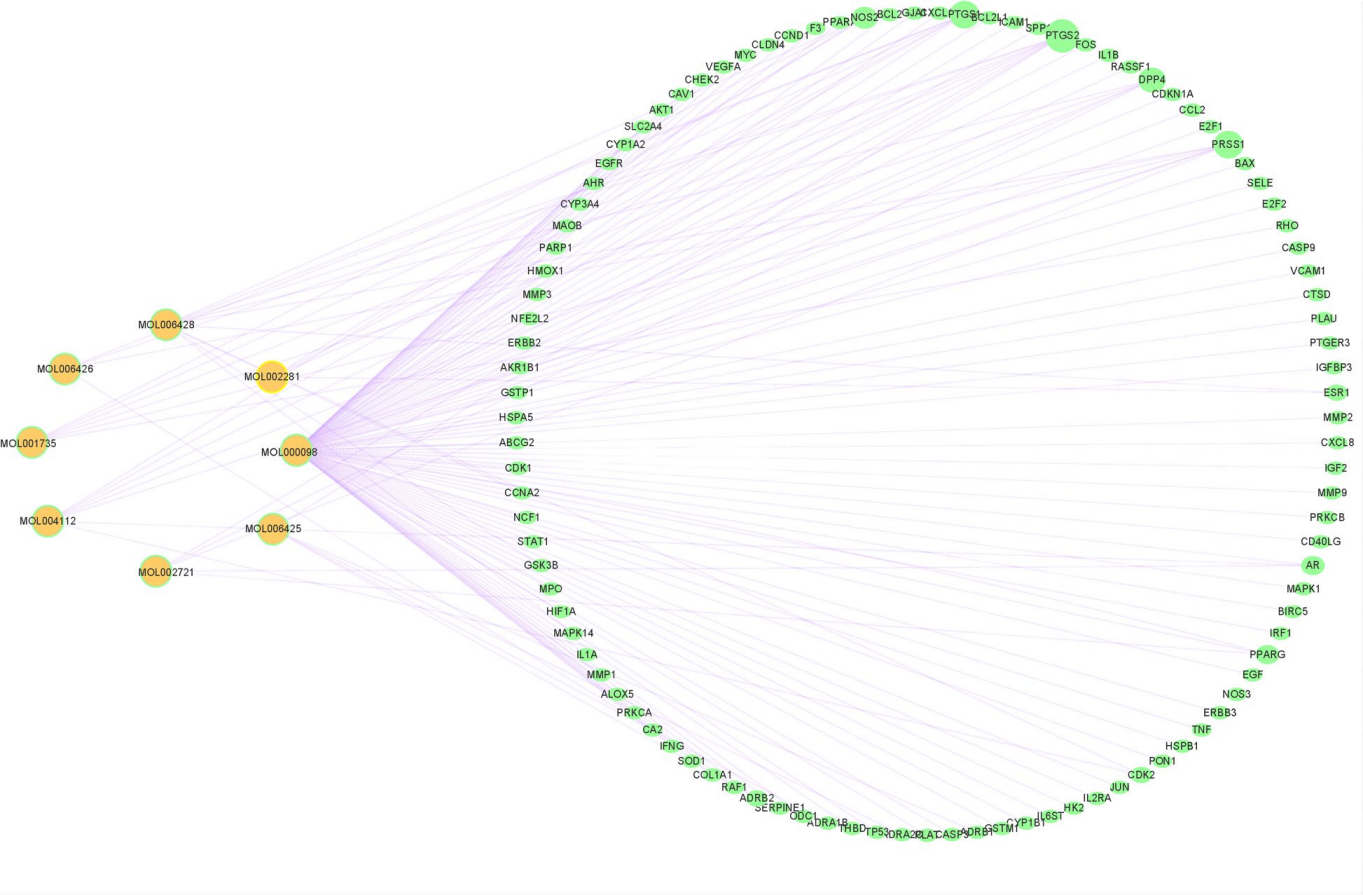
Compound-target network. The green circle nodes represent the targets of DR and *Eriocauli Flos*. The orange circle node represents the compound of *Eriocauli Flos*

The 8 compounds of *Eriocauli Flos* and 103 intersection targets were used to construct the compound-target network which consisted of 135 nodes (Fig. 2). Each edge represents the interaction of the compounds and targets. The total nodes and edges of the network were displayed in the Supplementary Table S1. And the network shows that one compound can associate with multiple compounds which accords with synthetic function of traditional Chinese medicine.

### PPI network of intersection targets of *Eriocauli Flos* and DR

**Fig. 3:**
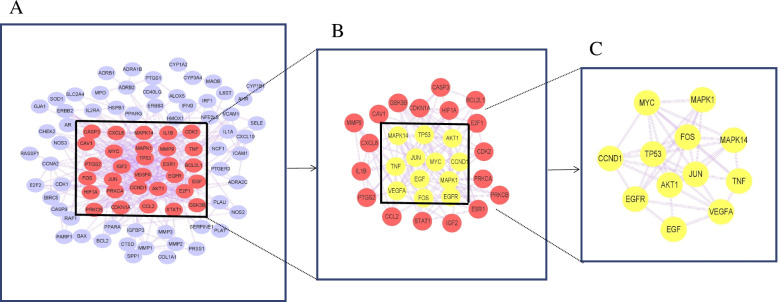
PPI network of intersection targets of *Eriocauli Flos* and DR**.** In terms of the filtering condition which CytoNCA scores are greater than the median value, the red circle represents the 30 genes screened for the first time **(A)**, the yellow circle represents the 12 genes obtained for the second time **(B)**, and the blue circle represents the unscreened genes **(C)**

One hundred three intersection targets were input to STRING database to build the PPI network and then the results of PPI network were imported to cytoscape for further analysis. The network was composed of 89 nodes and 612 edges. The top 12 key targets (TP53, MAPK14, CCND1, MAPK1, AKT1, EGFR, EGF, FOS, MYC, VEGFA, TNF-α and JUN) were narrowed down by betweenness, closeness and degree parameter of cytoNCA module (Fig. 3) [30]. The degree, betweenness and closeness of 12 core targets were listed in the Table 2.

**Table 2:** The major parameter of top 12 targets in PPI network of *Eriocauli Flos* and DR

Target	Betweenness	Closeness	Degree
TP53	82.66727347	0.707317073	34
MAPK14	30.98526381	0.58	22
CCND1	43.52465867	0.617021277	24
MAPK1	69.96781552	0.617021277	26
AKT1	89.88644596	0.69047619	32
EGFR	51.27220002	0.617021277	24
EGF	30.91583787	0.557692308	16
FOS	16.80648241	0.604166667	20
MYC	21.58809431	0.630434783	24
VEGFA	81.02487512	0.630434783	24
TNF	46.13921264	0.58	24
JUN	78.67520165	0.69047619	32

### GO and KEGG analysis of the intersection targets of *Eriocauli Flos* and DR

**Fig. 4:**
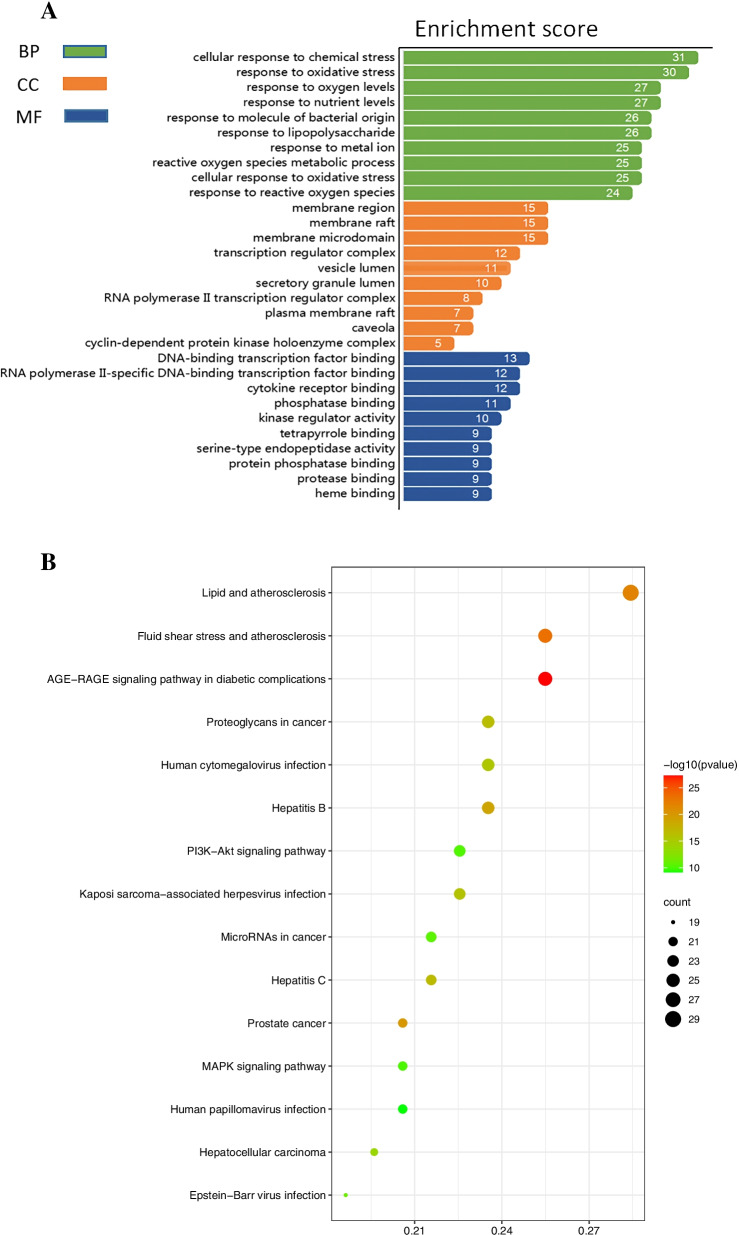
**A** GO analysis of intersection targets of *Eriocauli Flos* and DR (top 10 were listed). **B** KEGG analysis of intersection targets of *Eriocauli Flos* and DR (top 15 were listed)

**Fig. 5:**
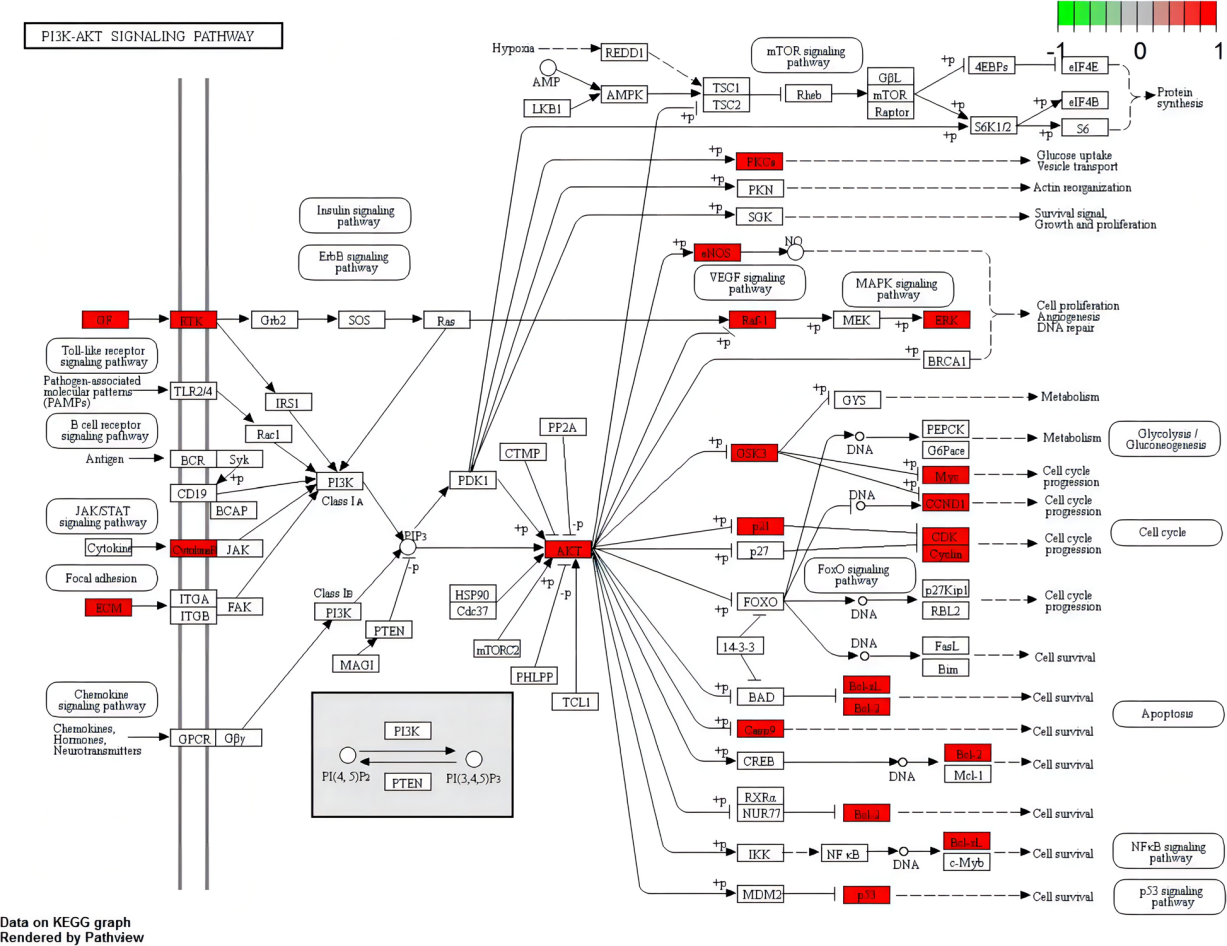
PI3K-Akt signaling pathway. The red rectangle represents proteins from the intersection targets of *Eriocauli Flos* and DR in the PPI network

A total 2248 enrichment results were obtained from 103 intersection targets which contained 2095 biological process (BP), 49 molecular functions (MFs) and 153 cellular components (CCs). The top 10 enrichment results of BP, MFs and CCs were listed in the Fig. 4A, and the most notable biological processes were enriched in cellular response to chemical stress, response to oxidative stress, response to reactive oxygen species, reactive oxygen species metabolic process and so on. The KEGG enrichment result indicated that the genes were mostly enriched in AGE-RAGE signaling pathway in diabetic complications (hsa04933), lipid atherosclerosis (hsa05417), fluid shear stress and atherosclerosis (hsa05418) and PI3K-Akt signaling pathway (hsa04151), as shown in the Fig. 4B. And the detailed information about PI3K-Akt signaling pathway related to DR was displayed in the Fig. 5.

### Molecular docking of the compounds and the core targets

**Fig. 6:**
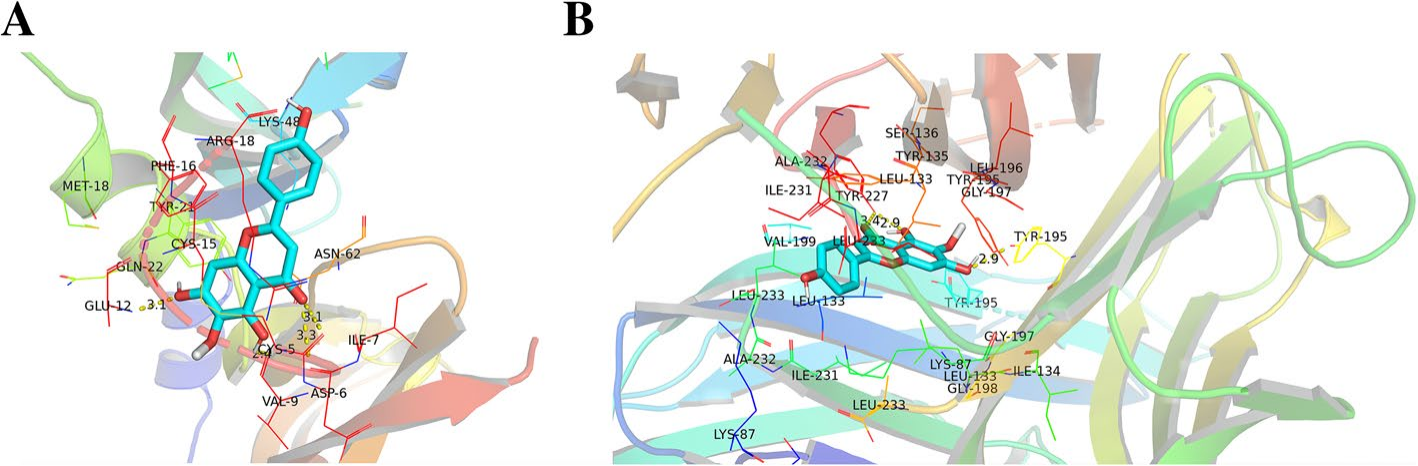
Representative 3D diagram of molecular docking. **A** Docking between Small molecule ligand hispidulin and target protein VEGFA. **B** Docking between Small molecule ligand hispidulin and target protein TNF-α

We selected VEGFA and TNF-α, from the core targets, which was closely related to DR, to dock with the 8 compounds. Generally, the value of vina scores exhibits the binding activity between a protein and a compound and the lower value represents greater affinity. The results indicated that hispidulin, among the 8 compounds, had the highest affinity with VEGFA and the second highest affinity with TNF-α, and the affinity of VEGFA to each component was higher than that of the original ligand (HT7) while the affinity of TNF-α to the original ligand (VGY) was opposite (Table 3). In addition, we found that hispidulin formed hydrogen bonds with CYS-5, ASP-6 and GLU-12 in VEGFA while having two with TYR-227 and one with TYR-195 in TNF-α. The representative 3D diagram of the docking results was shown in the Fig. 6.

**Table 3:** Vina scores of compound-target docking

Compound	VEGFA	TNF
1_3_6_trihydroxy_2_5_7_trimethoxyxanthone	−6.4	5.3
2_Benzo [1_3] dioxol_5_yl_5_7_dimethoxy	−6.2	9.9
7_30_dihydroxy_5_40_50_trimethoxyisoflavone	−6.3	−9.1
Dinatin (Hispidulin)	−6.6	9.4
Patuletin	−6.5	8.7
Quercetagetin	−6.6	8.4
Quercetin	−6.4	8.2
Toralactone	−6.5	7.1
HT7	−5.1	–
VGY	–	12.7

HT7 is the original ligand of VEGFA while VGY is the original ligand of TNF

### The effect of hispidulin on the proliferation in HRECs under high glucose condition

**Fig. 7:**
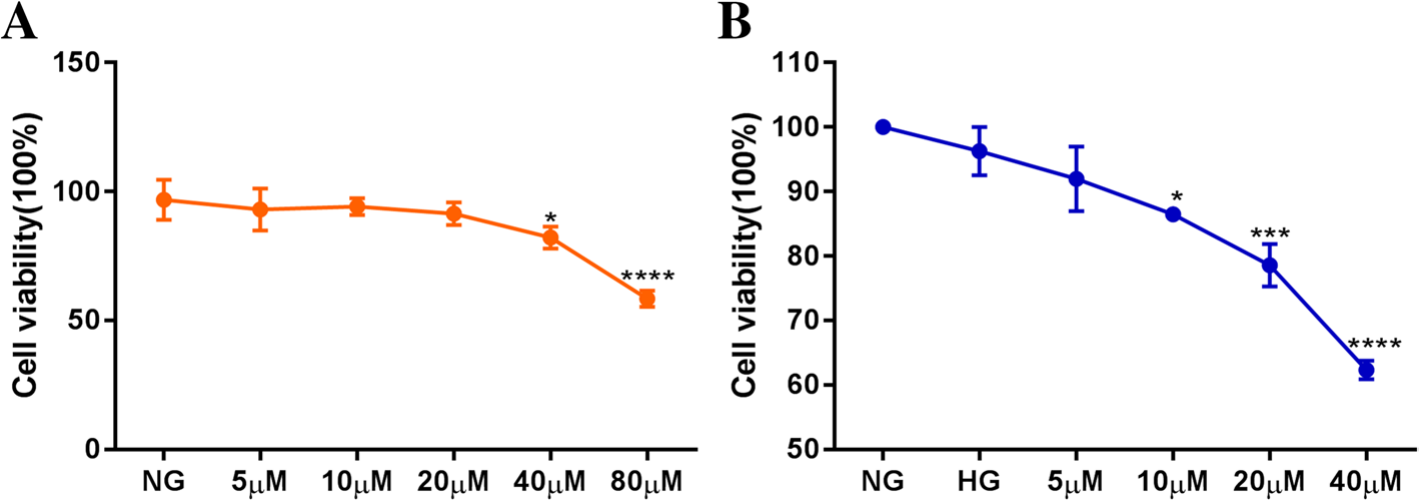
The cell viability of HRECs in the treatment of hispidulin with normal glucose or with high glucose. **A** The cytotoxicity of hispidulin on the HRECs under normal glucose (NG, 5 mM) condition for 24 h. Data were shown as mean ± SD (*n =* 3) and analyzed by one-way ANOVA and followed by Dunnett’s multiple comparison test. ^*^P < 0.05 and ^****^ *P* < 0.001 versus NG group. **B** The cell viability of hispidulin on the HRECs under high glucose (HG, 30 mM) condition for 48 h and the NG group was regarded as control group. Data were shown as mean ± SD (*n =* 3) and analyzed by one-way ANOVA and followed by Dunnett’ s multiple comparison test. ^*^P < 0.05, ^***^P < 0.001 and ^****^P < 0.001 versus HG group

To evaluate the effect of hispidulin cytotoxicity on HRECs, we measured the cell viability of HRECs under normal glucose condition (5 mmol/L *D*-glucose) for 24 h. As shown in the Fig. 7A, the cell viability was decreased after treatment with hispidulin over 40 μM. Then, the HRECs were incubated with various concentrations of hispidulin for 48 h with normal glucose (NG, 5 mmol/L) or high glucose (HG,30 mmol/L) and the cell proliferation rate was significantly decreased from 10 μM to 40 μM (Fig. 7B). Thus, 10 μM and 20 μM of hispidulin treated in HRECs for 48 h was selected in the subsequent experiments.

### The effect of hispidulin on the high glucose-induced VEGFA and TNF-α expression at the transcript level

**Fig. 8:**
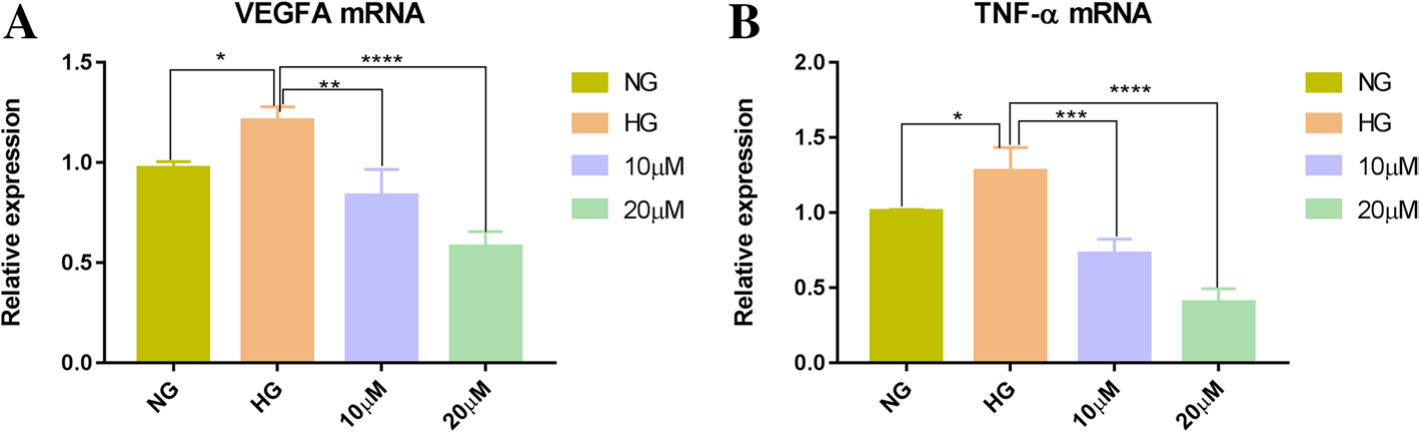
Effect of hispidulin on the mRNA expression of VEGFA **(A)** and TNF-α **(B)** under high glucose (HG, 30 mM) condition. Normal glucose (NG,5 mM) was regarded as control group and HG was model group. Data were shown as mean ± SD (*n =* 3) and analyzed by one-way ANOVA and followed by Dunnett’ s multiple comparison test. ^*^P < 0.05, ^**^P < 0.001, ^***^P < 0.001 and ^****^P < 0.001 versus HG group

To further examine the anti-inflammatory and anti-angiogenic effect of hispidulin on the regulation of VEGFA and TNF-α expression under high glucose conditions, we compared the transcription levels of VEGFA and TNF-α genes. The results demonstrated that the mRNA expression levels of VEGFA and TNF-α were increased after high glucose-stimulated HRECs culture for 48 h. Oppositely, the treatment of hispidulin significantly reduced the expression levels of VEGFA and TNF-α in a dose-dependent manner compared with high glucose group (Fig. 8).

### The effect of hispidulin on the high glucose-induced VEGFA and phosphorylation of Akt and ERK1/2 on protein level

**Fig. 9:**
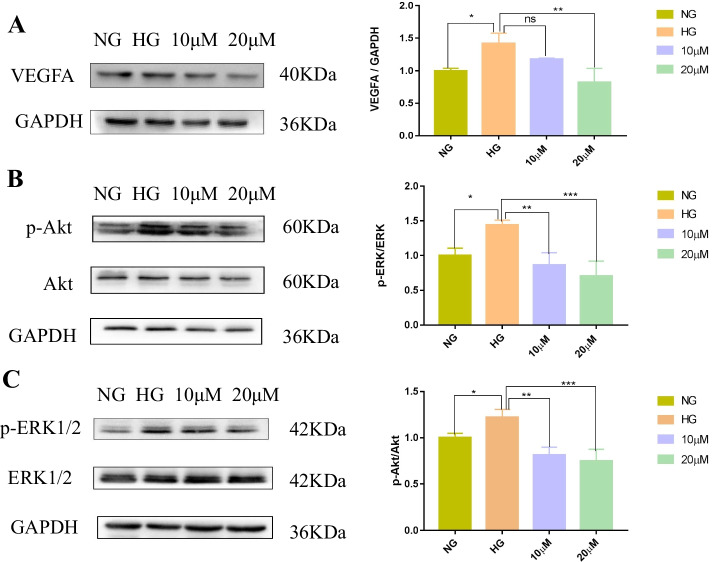
Effect of hispidulin on the protein expression level of VEGF (VEGFA) (A) and phosphorylation of Akt **(B)** and ERK1/2 **(C)** under high glucose (HG, 30 mM) condition. Normal glucose (NG,5 mM) was regarded as control group and HG was model group. In order to save and recycle antibodies and save time, the membrane was clipped into bands of corresponding molecular weight of proteins prior to hybridization with antibodies and the information was shown in the Supplementary Fig. S1. Data were shown as mean ± SD (*n =* 3) and analyzed by one-way ANOVA and followed by Dunnett’ s multiple comparison test. ^*^P < 0.05, ^**^P < 0.001 and ^***^P < 0.001 versus HG group

Through the network pharmacology analysis, the genes of VEGFA was predicted as the most crucial targets of *Eriocauli Flos* and DR. Furthermore, phosphorylation of Akt and ERK1/2 which play a vital role in the cell proliferation. Consequently, we found that treated with hispidulin showed down-regulating the expression of VEGFA only in the concentration of 20 μM, but decreasing phosphorylation of Akt and ERK1/2 in a dose-dependent manner (Fig. 9).

## Discussion

In this study, we employed a systematic network pharmacology method to explore the molecular mechanism of *Eriocauli Flos* in diabetic retinopathy. Through the PPI network and cytoNCA module analysis, two core genes VEGFA and TNF-α, were identified as major hub genes targets for DR, which has been paid to the key regulators in DR, [38] and also were validated as the biomarkers in our in vitro experiment. Furthermore, AKT1 and MAPK1 (ERK2), closely related to vascular endothelial proliferation, also existed in the core targets of PPI network which were tested in western blot experiment. Previous researches have proved that some proteins such as VEGFA, TNF-α, insulin-like growth factor 1 (IGF-1) and pigment epithelium-derived factor (PEDF) are responsible for the pathogenesis of DR, which contribute to retinal neovascularization in DR [39]. In addition, the results of GO enrichment analysis indicated that the genes were enriched in the response to oxidative stress and inflammation, which was also consistent with the pathological mechanism of DR [40, 41]. According to KEGG enrichment, *Eriocauli Flos* may modulate DR by regulating multiple pathways such as the AGE-RAGE and the PI3K-Akt signaling pathway in diabetic complications. Previous reports have indicated that the interaction between AGE and its receptor RAGE leaded to loss of retinal pericytes, and triggered inflammatory response and blood vessel formation [42]. In addition, VEGFA and VEGF receptor 2 (VEGFR2) have been implicated in direct activation through PI3K/Akt/eNOS pathway for endothelial cell proliferation, differentiation and angiogenesis [43]. Moreover, the results of molecular docking more deeply displayed the close interaction relationship between effective components and overlap targets. Particularly, hispidulin had a higher affinity with VEGFA and TNF-α which implied that it may have a potential therapeutic effect on DR. Hence, we selected hispidulin as the underlying target and selected VEGFA and TNF-α as the biomarkers of DR to perform the experimental validation. In our work, we build model by the treatment of 30 μM D-glucose for 48 h in HRECs in the light of previous research, [44] but in the aspect of cell proliferation, there was no change in high glucose group compared with normal glucose group which was possibly caused by glucose toxicity and VEGFA maybe the main factor in the regulation of proliferation [45] which provided a clue that VEGF and its downstream may regulates vascular endothelial cell proliferation through the PI3K-Akt signaling pathway. Thus, we measured the expression of VEGFA at transcription level and protein level, and our data showed that the expression of VEGFA significantly increased compared with normal glucose group, and the treatment of hispidulin counteract the effect while the VEGFA expression on protein level was not significant change in the concentration of 10 μM hispidulin which indicated that 20 μM hispidulin may be the optimal concentration in high glucose- induced HRECs. And the phenomena above conformed with the network analysis (PPI) and results of molecular docking. For verification, we performed western blot analysis for several representative members (p-Akt and p-ERK) of the PI3K-Akt pathway which was selected from the top 10 pathways of preliminary KEGG analysis. And the results in our experiment showed that hispidulin affected HRECs proliferation and VEGFA expression by inhibiting expression of p-Akt and p-ERK, which suggested that the anti-proliferation and anti-angiogenic effect of hispidulin on HRECs under high glucose condition was mediated by the inactivation of Akt1 and Erk1/2 signaling and the results were also in accord with the results of KEGG enrichment analysis. All in all, based on the analysis of network pharmacology and molecular docking, our study revealed that hispidulin may have anti-proliferation and anti-angiogenic effect on DR.

## Conclusion

Through network pharmacology analysis and by mining the data step by step, it was much clearer that *Eriocauli Flos* played a significant role in DR through multi-targets, multi-pathways approach. And hispidulin, a vital compound from *Eriocauli Flos,* exerts an anti-angiogenesis effect by down-regulating the expression of `p-AKT, p-ERK, and VEGF in vitro, therefore suggesting that hispidulin have potential as anti-VEGF agent in the therapy of DR. However, in our current study, it’s just simple verification of relevant targets and pathways in network pharmacology from the perspective of cell proliferation and the expression of VEGFA, and further in vivo studies are necessary to confirm the anti-angiogenic effect of hispidulin and to explore the underlying mechanisms of DR such as the effects on oxidative stress in terms of GO enrichment analysis.

## Supplementary Information

**Additional file 1.**

**Additional file 2.**

## Data Availability

The datasets used or analyzed during the current study are available from the corresponding author.
