# Correction: The potential effects and mechanisms of hispidulin in the treatment of diabetic retinopathy based on network pharmacology

**DOI:** 10.1186/s12906-022-03631-z

**Published:** 2022-06-08

**Authors:** Yao Chen, Jiaojiao Sun, Zhiyun Zhang, Xiaotong Liu, Qiaozhi Wang, Yang Yu

**Affiliations:** 1 Department of Histology Anatomy and HistoEmbryology, School of Basic Medical Sciences, Southwest Medical University, Luzhou, Sichuan 646000 P.R. China; 2 Department of Clinical Medicine, School of Clinical Medicine, Southwest Medical University, Luzhou, Sichuan 646000 P.R. China; 3 Jiangyang City Construction College, Luzhou, Sichuan 646000 P.R. China; 4 Key Laboratory of Medical Electrophysiology of Ministry of Education and Medical China, Luzhou, Sichuan 646000 P.R. China; 5 Electrophysiological Key Laboratory of Sichuan Province, Institute of Cardiovascular Research, Southwest Medical University, Luzhou, Sichuan 646000 P.R. China

**Correction: BMC Complement Med Ther 22, 141 (2022)**

**https://doi.org/10.1186/s12906-022-03593-2**

Following the publication of the original article [1], the authors reported that certain authors’ affiliations were assigned incorrectly.

The authors’ affiliations have been updated above and the original article [1] has been corrected.
