# Supplementary material for: The potential effects and mechanisms of hispidulin in the treatment of diabetic retinopathy based on network pharmacology
Source: BMC Complement Med Ther. 2022 May 19;22:141. doi: 10.1186/s12906-022-03593-2 (PMC9121581; doi:10.1186/s12906-022-03593-2)
Supplement: Supplementary file 1 — Additional file 1. [file 12906_2022_3593_MOESM1_ESM.pdf]

Supplementary Table S1. The nodes and edges of compound-target network.

Node1	Node2	Net	MolName
MOL001735	NOS2	target	Dinatin
MOL001735	PTGS1	target	Dinatin
MOL001735	PTGS2	target	Dinatin
MOL001735	DPP4	target	Dinatin
MOL001735	PRSS1	target	Dinatin
MOL001735	RHO	target	Dinatin
MOL002281	NOS2	target	Toralactone
MOL002281	PTGS1	target	Toralactone
MOL002281	ESR1	target	Toralactone
MOL002281	PTGS2	target	Toralactone
MOL002721	AR	target	quercetagetin
MOL002721	PPARG	target	quercetagetin
MOL002721	PTGS2	target	quercetagetin
MOL002721	DPP4	target	quercetagetin
MOL002721	PRSS1	target	quercetagetin
MOL004112	NOS2	target	Patuletin
MOL004112	PTGS1	target	Patuletin
MOL004112	AR	target	Patuletin
MOL004112	PTGS2	target	Patuletin
MOL004112	DPP4	target	Patuletin
MOL004112	CDK2	target	Patuletin
MOL004112	PRSS1	target	Patuletin
MOL006425	ADRB1	target	2-Benzo[1,3]dioxol-5-yl-5,7-dimethoxy-chroman
MOL006425	PTGS2	target	2-Benzo[1,3]dioxol-5-yl-5,7-dimethoxy-chroman
MOL006425	ADRA2C	target	2-Benzo[1,3]dioxol-5-yl-5,7-dimethoxy-chroman
MOL006425	ADRA1B	target	2-Benzo[1,3]dioxol-5-yl-5,7-dimethoxy-chroman
MOL006425	ADRB2	target	2-Benzo[1,3]dioxol-5-yl-5,7-dimethoxy-chroman

MOL006426	PTGS1	target	1,3,6-trihydroxy-2,5,7-trimethoxyxanthone
MOL006426	PTGS2	target	1,3,6-trihydroxy-2,5,7-trimethoxyxanthone
MOL006426	CA2	target	1,3,6-trihydroxy-2,5,7-trimethoxyxanthone
MOL006426	PRSS1	target	1,3,6-trihydroxy-2,5,7-trimethoxyxanthone
MOL006428	NOS2	target	7,30-dihydroxy-5,40,50-trimethoxyisoflavone
MOL006428	PTGS1	target	7,30-dihydroxy-5,40,50-trimethoxyisoflavone
MOL006428	ESR1	target	7,30-dihydroxy-5,40,50-trimethoxyisoflavone
MOL006428	PTGS2	target	7,30-dihydroxy-5,40,50-trimethoxyisoflavone
MOL006428	DPP4	target	7,30-dihydroxy-5,40,50-trimethoxyisoflavone
MOL006428	MAPK14	target	7,30-dihydroxy-5,40,50-trimethoxyisoflavone
MOL006428	GSK3B	target	7,30-dihydroxy-5,40,50-trimethoxyisoflavone
MOL006428	CDK2	target	7,30-dihydroxy-5,40,50-trimethoxyisoflavone
MOL006428	PRSS1	target	7,30-dihydroxy-5,40,50-trimethoxyisoflavone
MOL006428	CCNA2	target	7,30-dihydroxy-5,40,50-trimethoxyisoflavone
MOL000098	PTGS1	target	quercetin
MOL000098	AR	target	quercetin
MOL000098	PPARG	target	quercetin
MOL000098	PTGS2	target	quercetin
MOL000098	DPP4	target	quercetin
MOL000098	AKR1B1	target	quercetin
MOL000098	PRSS1	target	quercetin
MOL000098	ADRB2	target	quercetin
MOL000098	MMP3	target	quercetin
MOL000098	MAOB	target	quercetin
MOL000098	EGFR	target	quercetin
MOL000098	AKT1	target	quercetin
MOL000098	VEGFA	target	quercetin
MOL000098	CCND1	target	quercetin
MOL000098	BCL2	target	quercetin

MOL000098	BCL2L1	target	quercetin
MOL000098	FOS	target	quercetin
MOL000098	CDKN1A	target	quercetin
MOL000098	BAX	target	quercetin
MOL000098	CASP9	target	quercetin
MOL000098	PLAU	target	quercetin
MOL000098	MMP2	target	quercetin
MOL000098	MMP9	target	quercetin
MOL000098	MAPK1	target	quercetin
MOL000098	EGF	target	quercetin
MOL000098	TNF	target	quercetin
MOL000098	JUN	target	quercetin
MOL000098	IL6ST	target	quercetin
MOL000098	CASP3	target	quercetin
MOL000098	TP53	target	quercetin
MOL000098	ODC1	target	quercetin
MOL000098	RAF1	target	quercetin
MOL000098	SOD1	target	quercetin
MOL000098	PRKCA	target	quercetin
MOL000098	MMP1	target	quercetin
MOL000098	HIF1A	target	quercetin
MOL000098	STAT1	target	quercetin
MOL000098	CDK1	target	quercetin
MOL000098	HSPA5	target	quercetin
MOL000098	ERBB2	target	quercetin
MOL000098	HMOX1	target	quercetin
MOL000098	CYP3A4	target	quercetin
MOL000098	CYP1A2	target	quercetin
MOL000098	CAV1	target	quercetin

MOL000098	MYC	target	quercetin
MOL000098	F3	target	quercetin
MOL000098	GJA1	target	quercetin
MOL000098	ICAM1	target	quercetin
MOL000098	IL1B	target	quercetin
MOL000098	CCL2	target	quercetin
MOL000098	SELE	target	quercetin
MOL000098	VCAM1	target	quercetin
MOL000098	PTGER3	target	quercetin
MOL000098	CXCL8	target	quercetin
MOL000098	PRKCB	target	quercetin
MOL000098	BIRC5	target	quercetin
MOL000098	NOS3	target	quercetin
MOL000098	HSPB1	target	quercetin
MOL000098	IL2RA	target	quercetin
MOL000098	CYP1B1	target	quercetin
MOL000098	PLAT	target	quercetin
MOL000098	THBD	target	quercetin
MOL000098	SERPINE1	target	quercetin
MOL000098	COL1A1	target	quercetin
MOL000098	IFNG	target	quercetin
MOL000098	ALOX5	target	quercetin
MOL000098	IL1A	target	quercetin
MOL000098	MPO	target	quercetin
MOL000098	NCF1	target	quercetin
MOL000098	ABCG2	target	quercetin
MOL000098	GSTP1	target	quercetin
MOL000098	NFE2L2	target	quercetin
MOL000098	PARP1	target	quercetin

MOL000098	AHR	target	quercetin
MOL000098	SLC2A4	target	quercetin
MOL000098	CHEK2	target	quercetin
MOL000098	CLDN4	target	quercetin
MOL000098	PPARA	target	quercetin
MOL000098	CXCL10	target	quercetin
MOL000098	SPP1	target	quercetin
MOL000098	RASSF1	target	quercetin
MOL000098	E2F1	target	quercetin
MOL000098	E2F2	target	quercetin
MOL000098	CTSD	target	quercetin
MOL000098	IGFBP3	target	quercetin
MOL000098	IGF2	target	quercetin
MOL000098	CD40LG	target	quercetin
MOL000098	IRF1	target	quercetin
MOL000098	ERBB3	target	quercetin
MOL000098	PON1	target	quercetin
MOL000098	HK2	target	quercetin
MOL000098	GSTM1	target	quercetin
