# Supplementary material for: The potential effects and mechanisms of hispidulin in the treatment of diabetic retinopathy based on network pharmacology
Source: BMC Complement Med Ther. 2022 May 19;22:141. doi: 10.1186/s12906-022-03593-2 (PMC9121581; doi:10.1186/s12906-022-03593-2)
Supplement: Supplementary file 2 — Additional file 2. [file 12906_2022_3593_MOESM2_ESM.pdf]

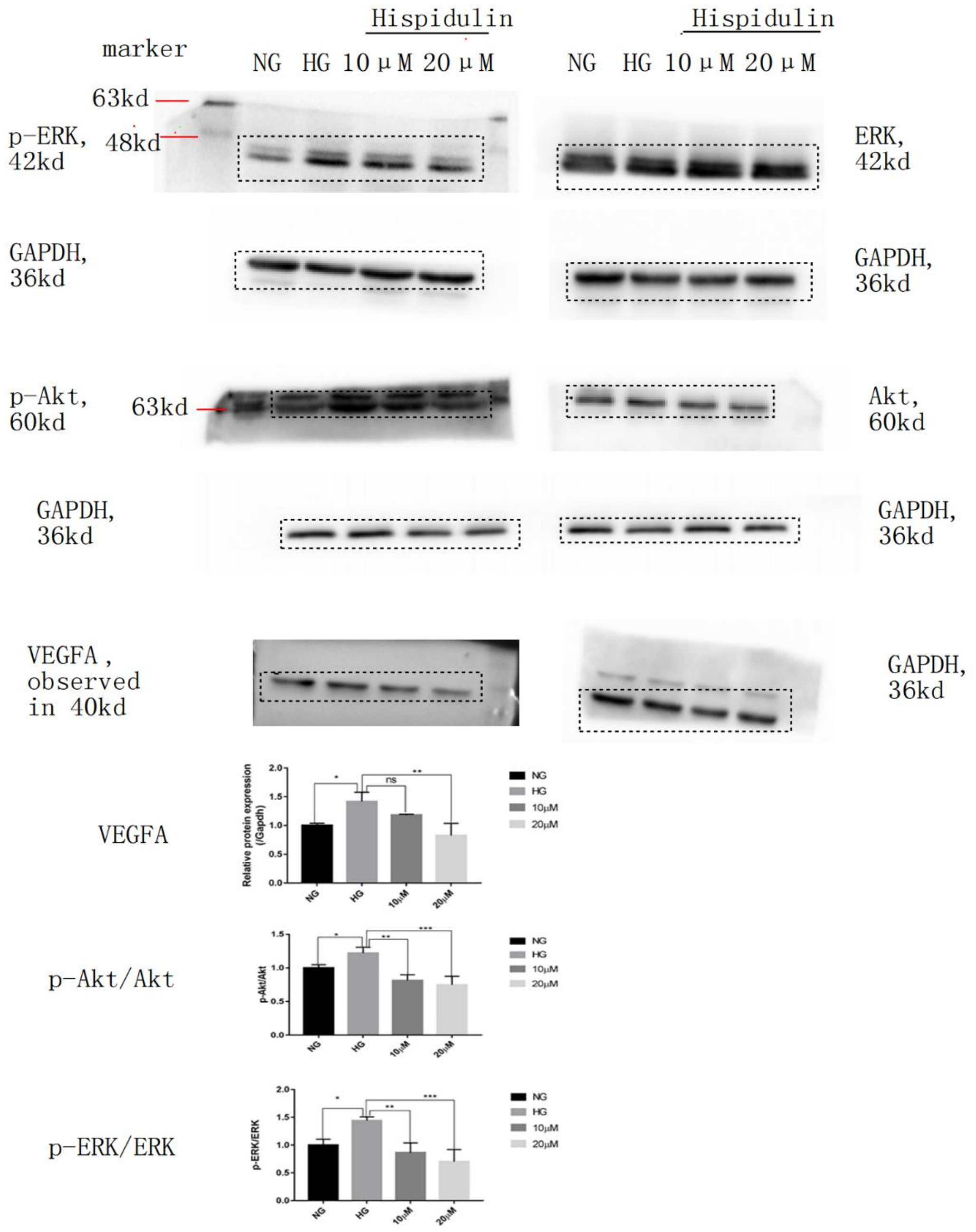

Supplementary Figure S1: Effect of hispidulin on the protein expression level of VEGF (VEGFA) and phosphorylation of Akt and ERK1/2 under high glucose (HG, 30mM) condition.

A. Red line showed the site of marker (PR1910, Solarbio) and the marker's color of blots under 48kd was not obvious which was possibly due to overexposure.

B. The dashed boxes represented the part of clipped blots used in the main text of manuscript.

C. The results showed that only 20μM concentration of hispidulin down-regulated the expression of VEGFA, but both 10μM and 20μM concentration of hispidulin decreased phosphorylation of Akt and ERK1/2 which was in a dose-dependent manner.

D. Data are presented as mean ± SD (n=3) and analyzed by one-way ANOVA and followed by Dunnett's multiple comparison test. *P<0.05, **P<0.01 and ***P <0.001 versus HG group.

All the original blots for all replicates performed.

The sample group: Hispidulin

NG HG 10μM 20μM

p-ERK **ERK**

GAPDH **GAPDH**

1.

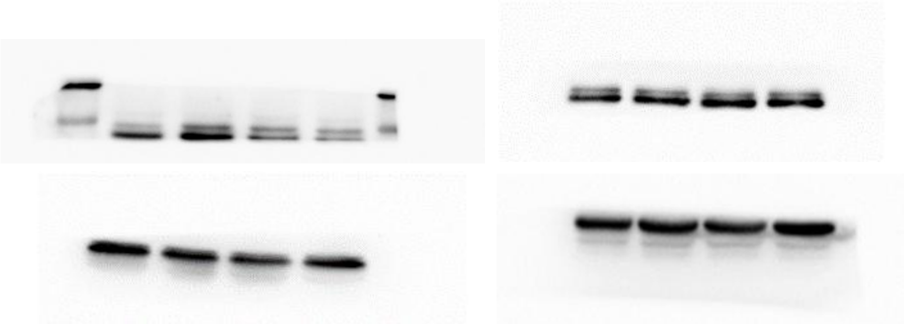

2.

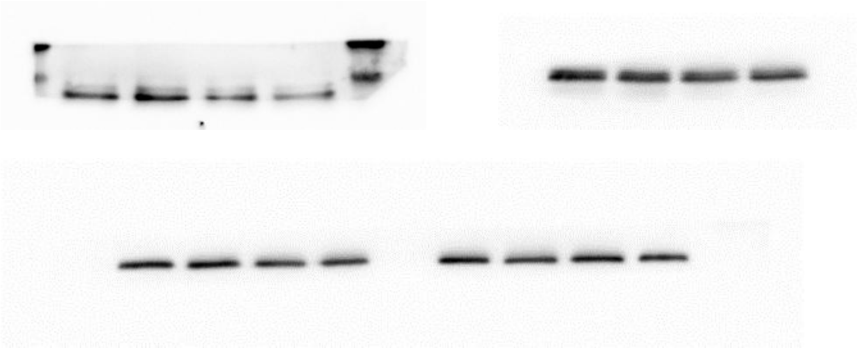

3. This is the original blots of p-ERK/ERK/GAPDH in the manuscript

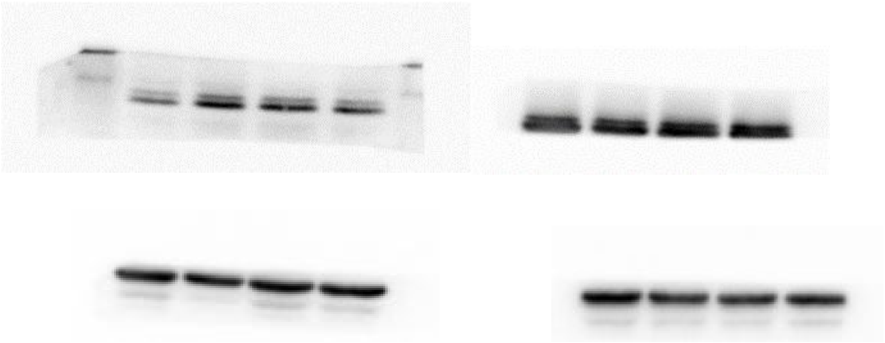

p-Akt **Akt**

GAPDH **GAPDH**

1.

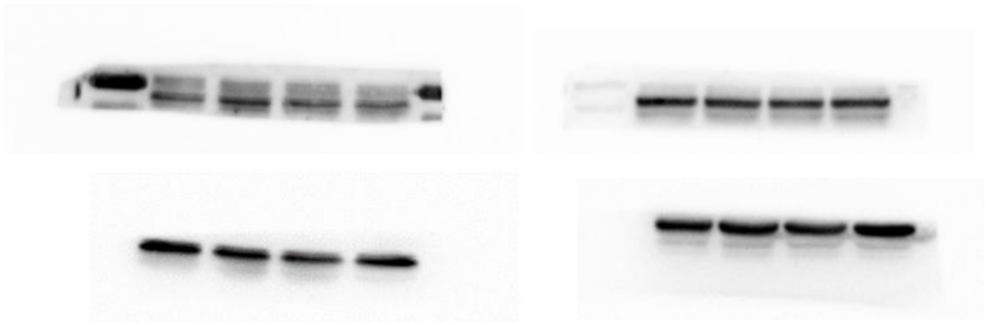

2. This is the original blots of p-Akt/Akt/GAPDH in the manuscript

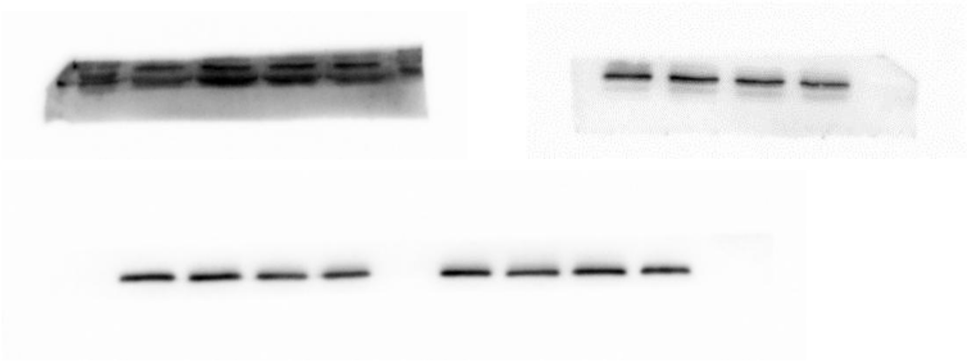

3.

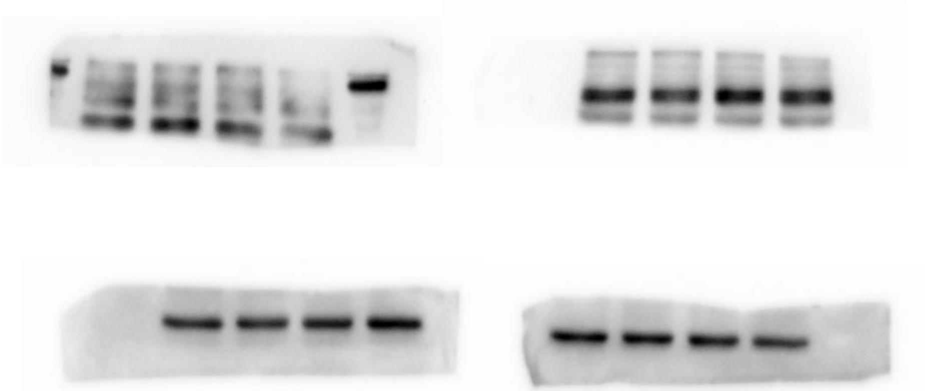

VEGFA **GAPDH**

1.

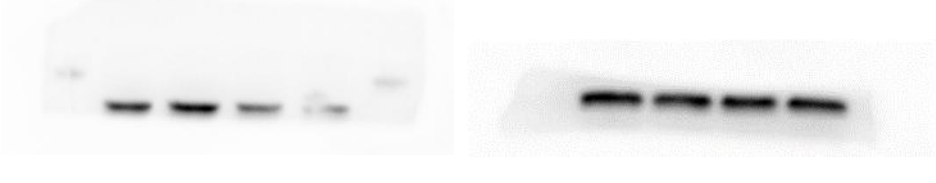

2.

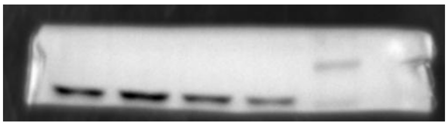

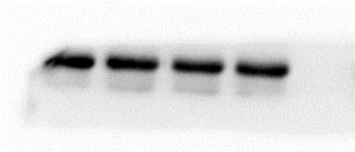

3. This is the original blots of VEGFA/GAPDH in the manuscript

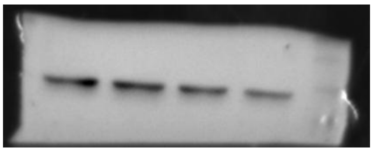

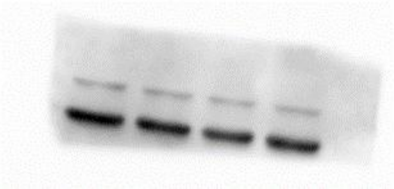

Note: the gapdh blots of the first two picture were clipped and the antibody of last gapdh was diretly incubated after the left blot of VEGFA.
